# Morphological and molecular characterization of invasive *Biomphalaria straminea* in southern China

**DOI:** 10.1186/s40249-018-0505-5

**Published:** 2018-12-08

**Authors:** Mohamed R. Habib, Shan Lv, Yun-Hai Guo, Wen-Biao Gu, Claire J. Standley, Roberta L. Caldeira, Xiao-Nong Zhou

**Affiliations:** 1 Medical Malacology Laboratory, Theodor Bilharz Research Institute, Giza, 12411 Egypt; 2 National Institute of Parasitic Diseases, Chinese Center for Diseases Control and Prevention; Key Laboratory of Parasite and Vector Biology, Ministry of Health, Shanghai, 200025 China; 3 Chinese Center for Tropical Diseases Research; WHO Collaborating Centre for Tropical Diseases; National Center for International Research on Tropical Diseases, Ministry of Science and Technology, Shanghai, 200025 China; 4 Milken Institute, School of Public Health, George Washington University, Washington, D.C, 20052 USA; 5 Grupo de Pesquisas em Helmintologia e Malacologia Médica, Instituto René Rachou/Fiocruz, Av. Augusto de Lima, Belo Horizonte, MG 1715 Brazil

**Keywords:** *Biomphalaria straminea*, Molecular taxonomy, *Schistosoma mansoni*, Intestinal schistosomiasis, Susceptibility, Invasive species

## Abstract

**Background:**

Schistosomiasis is a common parasitic disease designated as a neglected tropical disease by the World Health Organization. Schistosomiasis mansoni is a form of the disease that is caused by the digenean trematode *Schistosoma mansoni*, transmitted through *Biomphalaria* spp. as an intermediate host. *Biomphalaria* was introduced to Hong Kong, China in aquatic plants shipments coming from Brazil and the snail rapidly established its habitats in southern China. Earlier studies of *Biomphalaria* spp. introduced to southern China identified the snails as *Biomphalaria straminea*, one of the susceptible species implicated in *S. mansoni* transmission in South America. However, recent molecular investigations also indicated the presence of another South American species, *B. kuhniana,* which is refractory to infection. As such, it is important to identify accurately the species currently distributed in southern China, especially with emerging reports of active *S. mansoni* infections in Chinese workers returning from Africa.

**Methods:**

We combined morphological and molecular taxonomy tools to precisely identify *Biomphalaria* spp. distributed in Guangdong Province, southern China. In order to clearly understand the molecular profile of the species, we constructed a phylogeny using mtDNA data (COI and 16S rRNA sequences) from six populations of *Biomphalaria* spp. from Shenzhen City in Guangdong Province. In addition, we examined the external morphology of the shell and internal anatomy of the reproductive organs.

**Results:**

Both morphological and molecular evidences indicated a close affinity between *Biomphalaria* spp. populations from Guangdong and *B. straminea* from Brazil. The shell morphology was roughly identical in all the populations collected with rounded whorls on one side and subangulated on the other, a smooth periphery, an egg-shaped aperture bowed to one side, and a deep umbilicus. The shape and number of prostate diverticula (ranged from 11.67 to 17.67) in Guangdong populations supports its close affinity to *B. straminea* rather than *B. kuhniana.* Molecular analysis did not conflict with morphological analysis. Little genetic differentiation was observed within *Biomphalaria* populations collected. Phylogenetic analysis of COI and 16S rRNA haplotypes from snails collected and *B. straminea* sequences from Brazil and China using Bayesian inference revealed that Guangdong populations were clustered in one clade with *B. straminea* from Hong Kong of China and *B. straminea* from Brazil indicating their close affinity to each other.

**Conclusions:**

Data obtained in the current study clearly show that the populations of *Biomphalaria* spp. investigated are *B. straminea,* and we assume that those snails were either introduced via passive dispersal from Hong Kong of China or as a result of multiple introduction routes from Brazil.

**Electronic supplementary material:**

The online version of this article (10.1186/s40249-018-0505-5) contains supplementary material, which is available to authorized users.

## Multilingual abstract

Please see Additional file 1 for translations of the abstract into the five official working languages of the United Nations.

## Background

Schistosomiasis is an endemic parasitic disease affecting almost 240 million people worldwide, and an additional 700 million people are at risk of infection. The infection is prevalent in seventy-eight developing, tropical and sub-tropical countries [1, 2]. Some species of the genus *Biomphalaria* act as obligatory intermediate hosts for *Schistosoma mansoni* Sambon, 1907, the parasite responsible for intestinal schistosomiasis in different parts of the world. In the absence of either the parasite or the snails, the life cycle cannot be maintained [3, 4]. Efforts are thus directed toward eradicating intermediate hosts using molluscicides and/or the parasite via treatment of infected populations using praziquantel (the drug of choice for schistosomiasis treatment) [5–8]. *Biomphalaria* species have a close association with freshwater habitats closely related to human activities [9, 10]. The natural histories of the various species of *Biomphalaria* snails have been studied extensively, although taxonomic difficulties and population-level variation are among the factors which have confounded this task [5]. A detailed knowledge of the geographic distribution of *Biomphalaria* snails is important for the control of intestinal schistosomiasis and its epidemiologic surveillance [11]. *Biomphalaria* snails are thought to have evolved in South America and are currently distributed throughout the continent as well as in central America, the Caribbean, Africa and the Middle East [12, 13]. In addition to South America and Africa, *Biomphalaria* spp. was also reported from Asia in Hong Kong, China 40 years ago [14], as a result of human-mediated accidental introduction, likely via aquatic plants imported from Brazil for commercial use [15]. The introduced snail was identified as *B. straminea* (Dunker, 1848)*,* which is known by its invasiveness and colonization capabilities [16].

In China, in the years following its introduction, *B. straminea* dispersed through different watercourses in southern China. The snail was identified in numerous water bodies in Shenzhen City, Guangdong Province, China in 1981 [17], and in the next 2 years it showed a wider distribution in Shenzhen rivers, suggesting an origination from Hong Kong’s river system [18]. This distribution pattern confirms the ability of *B. straminea* to survive and form new colonies in southern China and its ability to spread to new water bodies [19]. The continuous rising in Earth’s surface temperature and climate change [20], as well as the continued rise in the mean annual temperature of China [21], may accelerate *B. straminea* spread due to its ability to adapt to different climate and ecological conditions [22]. In addition, the risks for *B. straminea* to be the source of *S. mansoni* transmission has been evaluated with the following concerns: (i) the involvement of China in numerous projects in Africa and the export of labor services to regions endemic with African schistosomiasis; (ii) *S. mansoni* infections have been reported in Chinese workers returning from Africa [23]; (iii) The presence of snails and infected humans in the same settings will increase the potential of *S. mansoni* transmission to be established in southern China in view of the role played by environmental changes such as climate change and increase in water manipulation and intensification of irrigation [23, 24].

A multidisciplinary approach for identification of species of medical importance combining morphological and molecular techniques is fundamental for our understanding of diseases epidemiology and surveillance. Proper identification of the intermediate host will help to map the potential schistosomiasis transmission foci in endemic areas as well as in areas without the disease but with abundant susceptible species [25]. For example, although *Biomphalaria* snails found in southern China were referred to as *B. straminea*, a new challenge was encountered. In 2015 a morphological and molecular study for seven *Biomphalaria* populations from Guangdong Province, China showed that, five of the populations are likely to be *Biomphalaria kuhniana* (Clessin, 1883) but not *B. straminea* [26]. The study suggested multiple colonization events and displacement of *B. straminea* by the closely related species, *B. kuhniana*. The latter species is also a neotropical snail described from Suriname, but reported in other South American countries [27]. Together, *B. straminea*, *B. kuhniana*, and *B. intermedia* (Paraense and Deslandes, 1962), are known as *B. straminea* complex [28]. The previous three species differ greatly in their susceptibility to infection with *S. mansoni*; for example, *B. kuhniana* is proven to be refractory to infection [27]. Thus, an urgent taxonomy of these species should be carefully examined through combined analysis of morphological and molecular variables, and their biomedical importance in the transmission of *S. mansoni* should be evaluated. Careful identification of *Biomphalaria* snails in southern China will lead to a better delineation based on biological profiles related to the potential transmission risk, and will help to develop an appropriate intervention for schistosomiasis surveillance or control in potential foci, particularly in areas of constrained resources.

## Methods

### Snail sampling and collection sites

**Fig. 1:**
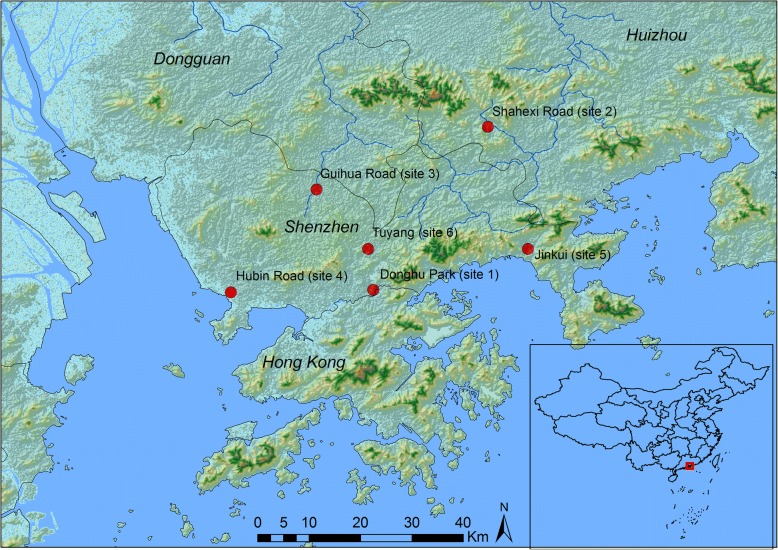
Map of the surveyed sites for *Biomphalaria* snails in the South of China

*Biomphalaria* snails were collected from six locations in Shenzhen during April and May, 2015 (Fig. 1). Sampling was quantified, with two collectors inspecting a 50–100 m length of the water body for 30 min and collecting all *Biomphalaria* found. A snail dip net with long handle was used for snail collection. Sampling was carried out at different water levels [29]. The materials recovered by the nets were examined for *Biomphalaria* snails, which were carefully collected with long blunt forceps and kept in plastic jars containing water from the same watercourse. Species identifications in the field were made through shell morphology, based on published keys [5, 30, 31]. As presented in Table 1, other gastropods were identified at species level [26, 32] and their presence recorded.

**Table 1:** Habitats, number of snails and GPS coordinates for snails’ localities

Locality name	Region	Drainage system	Coordinates	Number of collected *Biomphalaria* (Planorbidae)	Other freshwater gastropods	Substrate
Donghu Park	Luohu District, Shenzhen	Shenzhen reservoir	N22.554° E114.149 ° H 12 M	500	Ampullariidae: *Pomacea canaliculata* Thiaridae: *Melanoides tuberculata* Physidae: *Physella acuta* Lymnaeidae: *Radix auriculariaplicatula* Viviparidae: *Bellamya chinensis*	MR
Shahexi Road	NanshanDistrict, Shenzhen	Dasha River	N22.84° E114.35° H 14 M	100	Ampullariidae: *Pomacea canaliculata* Thiaridae: *Melanoides tuberculata* Physidae: *Physella acuta* Lymnaeidae: *Radix auriculariaplicatula*	SM
Guihua Road	Longhua District, Shenzhen	Guanlan River	N22.73 N 114.05° H 10 M	70	Ampullariidae: *Pomacea canaliculata* Physidae: *Physella acuta*	S
Hubin Road	Baoan District, Shenzhen	Xinzhen River	N22.55° E113.90° H 3.6 M	50	Ampullariidae: *Pomacea canaliculata* Thiaridae: *Melanoides tuberculata* Physidae: *Physella acuta*	SR
Jinkui	Dapeng District, Shenzhen	Kuichong River	22.626 N 114.420 H 5 M	350	Ampullariidae: *Pomacea canaliculata* Physidae: *Physella acuta*	SR
Tuyang	Dapeng District, Shenzhen	Kuichong River	22.626 N 114.140 H 5 M	850	Physidae: *Physella acuta*	M

*M* mud, *MR mud+rocks, S* sands, *SR* sands-rocks, *SM* sands+mud

Each location was georeferenced with a handheld geographical positioning systems (GPS) device (Garmin GPSMap 60CS, Garmin Ltd., Kansas City, USA) and the coordinates were recorded. The in situ water temperature (°C), microconductivity (measured in micro-siemens, μS), and pH, all factors that have been implicated as potentially important factors influencing freshwater snail distribution, were recorded [5, 33]. These water chemistry variables were measured using a PH-200 handheld portable pH and temperature meter and AP-2 aquapro conductivity meter (HM Digital, Inc., Culver City, USA). The substrate type was also observed. Collected *Biomphalaria* snails were sorted per site after returning to the laboratory. The snails were placed in individual wells filled with 2 ml of dechlorinated water and exposed to artificial light for 3 h to encourage emergence of trematodes cercariae. After exposure, the wells were inspected with a dissecting microscope for the presence of infection.

### Identification of collected *Biomphalaria* snails

#### Morphological identification

Ten snails from each site were allowed to relax overnight in an aqueous solution of menthol, followed by rinsing in water heated at 70 °C for 40 s. The snails were then removed from their shells individually and the head-foot was cut off and placed in 75% ethanol for molecular work and the remaining soft tissue was placed in a modified Raillet–Henry fluid (distilled water 930 ml, sodium chloride 6 g, 40% formalin 50 ml, and glacial acetic acid 20 ml) [34]. Shells were cleaned with 0.5% sodium hypochlorite (CLOROX), and photographed using an AxioCam attached to a stereomicroscope (Olympus SZX12, Japan). Two sets of morphological measurements were taken: conchological and internal anatomical measurements. The number of whorls was counted using a hand held magnifying lens. The shell height, shell diameter, diameter of the umbilicus, ratio between shell height and diameter were measured using digital calipers (accurate to 0.01 mm). The soft tissue preserved in Raillet–Henry fluid (5 per site) was dissected to reveal the internal anatomy of each snail, and particularly the reproductive organs. After dissection of the reproductive system under the stereoscopic microscope, three measurements were made: length of penis sheath, length of preputium and number of prostate diverticula. Mean ratios between shell height and shell diameter and length of penis sheath and length of preputium were compared by an analysis of variance (ANOVA) and Scheffé test [35]. Differences were considered significant at *P* < 0.05.

#### Molecular characterization of collected snails

Genomic DNA was extracted separately from 10 head-foot samples preserved in ethanol using DNeasy blood and tissue kit (Cat No. 69506, QIAGEN GmbH, Hilden, Germany) following the manufacturer instructions and stored at 4 °C. Polymerase chain reaction (PCR) amplifications were made using Bio-Rad C1000 thermal cycler (Bio-Rad, USA). Two regions of the mitochondrial genome; the cytochrome oxidase subunit I (COI) gene and the 16S sub-unit of ribosomal RNA (16S rRNA) were amplified. The general PCR cycle used was 2 min initial denaturing at 94 °C, followed by 35 cycles of 1 min at 94 °C, 45 s at the annealing temperature and 1 min extension at 72 °C; primer details and annealing temperatures can be found in Table 2 [13, 36, 37]. PCR products were visualized on a 2% agarose gel stained with Goldview (No. 141008, SBS, China). The PCR products were then sent to Sangong Biotech Company (Shanghai, China) for sequencing.

**Table 2:** Primer sets with sequences for amplification of cytochrome oxidase I (COI) and 16S rRNA

Primer name	Region	Sequence (5′ → 3′)	Annealing temperature (°C)
LCO1490	COI (sense)	GGTCAACAAATCATAAAGATATTGG	45
HCO2198	COI (antisense)	TAAACTTCAGGGTGACCAAAAAATCA	45
16arm	16S rRNA (sense)	CTTCTCGACTGTTTATCAAAAACA	50
16brm	16S rRNA (antisense)	GCCGGTCTGAACTCAGATCAT	50

#### Sequences alignment and genetic analysis

Nucleotide sequences were visually edited using Bioedit software v 7.0 [38]. BLAST searches were performed for the obtained sequences at NCBI (http://www.ncbi.nlm.nih.gov/) against the GenBank database for initial identification of the collected snails. The sequences were aligned using Clustal X [39]. The COI and 16S rRNA data sets were phylogenetically analyzed separately and as a combined data set. DNA samples of *B. straminea*, *B. kuhniana*, and *B. intermedia* (*B. straminea* complex) from some Brazilian states and from Colombia were obtained from Medical Malacological Collection (Fiocruz-CMM) of René Rachou Institution. The 16S rRNA gene was sequenced from these samples and used in the phylogeny construction of the 16S rRNA tree. Unfortunately, the COI gene could not be sequenced as most of the DNA was lost during the shipment process and the remaining amount was only sufficient for testing one genetic region (16S rRNA). The alignments of all data sets were based on gap-free sequences. Phylogenetic relationships were estimated using Bayesian inference in MrBayes version 3.2.0 programs [40], using haplotypes from the current study and all known sequences of *B. straminea* and *B. kuhniana* from GenBank. *Biomphalaria tenagophila* (Orbigny, 1835) was chosen as an out-group. Prior to Bayesian inference, the best fit nucleotide substitution models (HKY for COI and for combined data set and TrN for 16S rRNA) were determined using a hierarchical likelihood ratio test in jMODELTEST version 0.1.1 [41]. The posterior probabilities were calculated via 6 000 000 generations using Markov chain Monte Carlo (MCMC) simulations, and the chains were sampled every 1000 generations. At the end of this run, the average standard deviation of split frequencies was below 0.01, and the potential scale reduction factor was reasonably close to 1.0 for all parameters. A consensus tree was summarized and visualized in FigTree version 1.4.3 (http://tree.bio.ed.ac.uk/software/figtree/). Evolutionary analyses were conducted in MEGA6 [42] using the Maximum Composite Likelihood model and codon positions included were 1st + 2nd + 3rd + Noncoding. All positions containing gaps and missing data were eliminated.

## Results

### Collection of snails and study area

Our malacological surveys included six separate locations in Shenzhen City (Fig. 1 and Table 1). All the locations were typical habitats for snails and all were positive for the presence of *Biomphalaria* with variable population densities with numbers ranging from 50 snails as in Hubin, Xinzhen River to 850 snails in Tuyang, Kuichong River. The largest numbers were found near the streams’ banks crawling on substrates or attached to aquatic plants. The depth of the habitats was qualitatively moderate (10–50 cm). No significant differences were observed in the physiochemical properties of water bodies harboring the snails. The water temperature ranged from 27 to 29 °C, pH from 7.4 to 8, and conductivity from 190 to 570 μS. The total dissolved solids were within the range of 0.18–0.28 parts per thousand (ppt). The type of the substratum was variable from one habitat to another. The substrate was formed of mud, sand, rock or a combination of these substrates. The most common freshwater snails co-localized in most of the habitats were *Pomacea canaliculata* (Lamarck, 1828) and *Physella acuta* (Draparnaud, 1805). The screening of collected snails for natural trematode infections revealed the absence of any trematode larvae.

### Morphology

**Fig. 2:**
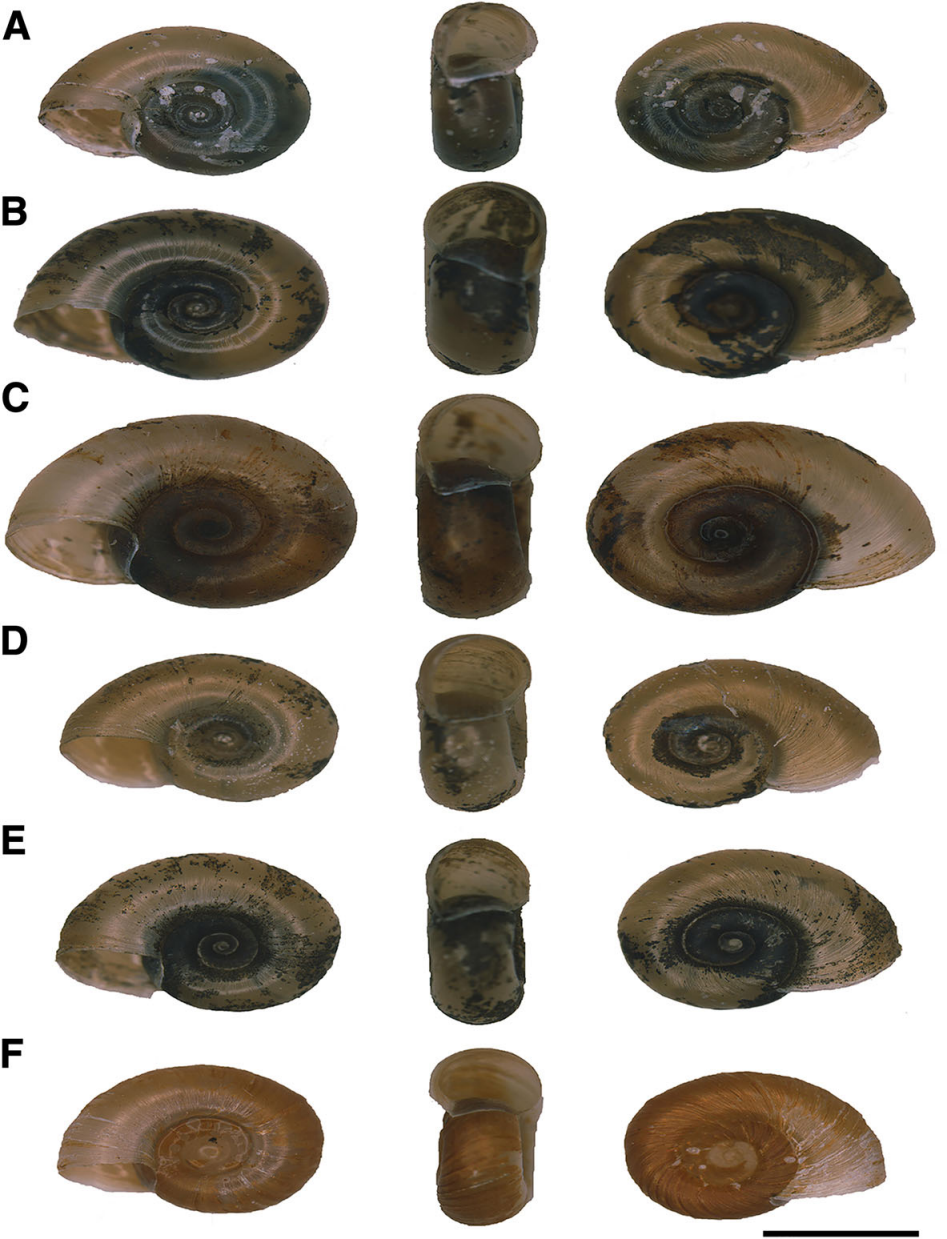
Shells of *Biomphalaria straminea*. Collected from different localities in Guangdong Province. **a**: Donghu Park, **b**: Shahexi Road, **c**: Guihua Road, **d**: Hubin Road, **e**: Jinkui, **f**: Tuyang. Bar = 5 mm

Shell characteristics were roughly the same in most of the collected *Biomphalaria* snails, with rounded whorls on one side and subangulated on the other, a smooth periphery, an egg-shaped aperture bowed to one side, and a deep umbilicus. Most of the snails shared the same number of whorls (4 whorls). Shell measurements were variable among the populations, with significant differences in overall length and height (*P* < 0.001 by ANOVA) among the different populations though no significant differences were observed in the length to height ratio (Fig. 2 and Table 3).

**Table 3:** Measurements of shell and reproductive organs of *Biomphalaria straminea* collected from different localities in southern China (Mean ± SD)

Locality name	Shell					Anatomy	
	Diameter	Height	Height/Diameter	Aperture height	Spire diameter	PS length/PP length	PD
Donghu Park	7.59 ± 0.45	2.96 ± 0.14	0.39 ± 0.02	3.07 ± 0.18	2.82 ± 0.28	1.24 ± 0.08	20.6 ± 2.3
Shahexi Road	9.16 ± 0.75	3.51 ± 0.26	0.38 ± 0.02	3.70 ± 0.33	3.37 ± 0.44	1.32 ± 0.17	15.2 ± 2.88
Guihua Road	9.70 ± 0.93	3.45 ± 0.34	0.36 ± 0.02	3.73 ± 0.30	3.45 ± 0.50	1.24 ± 0.12	13.4 ± 0.89
Hubin Road	8.24 ± 0.38	3.03 ± 0.14	0.37 ± 0.02	3.31 ± 0.23	2.80 ± 0.37	1.35 ± 0.10	12.2 ± 1.4
Jinkui	8.36 ± 0.30	3.04 ± 0.14	0.36 ± 0.01	3.40 ± 0.14	2.69 ± 0.26	1.41 ± 0.28	10.8 ± 0.83
Tuyang	8.41 ± 0.40	2.97 ± 0.21	0.35 ± 0.02	3.48 ± 0.24	3.16 ± 0.33	1.32 ± 0.19	14.8 ± 0.83

*PS* Penis sheath, *PP* Preputium, *PD* Prostate diverticula

**Fig. 3:**
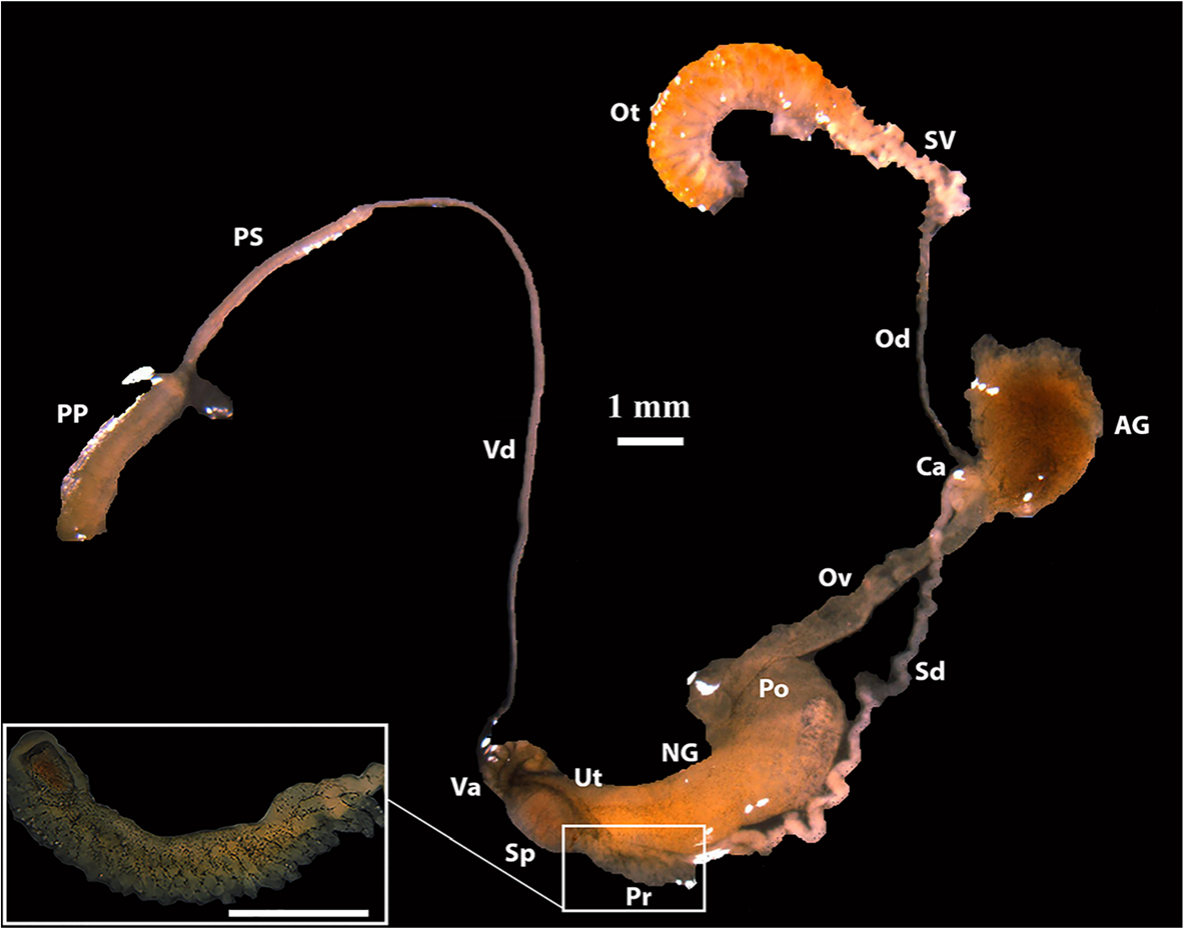
Anatomy of the reproductive system of *Biomphalaria straminea*, Tuyang, Shenzhen, Guandong Province. AG, albumin gland; Ca, carrefour; NG, nidamental gland; Od, ovispermiduct; Ot, ovotestis; Ov, oviduct; Po, pouch of the oviduct; PP, preputium; Pr, prostate; PS, penis sheath; Sd, spermduct; Sp, spermatheca; SV, seminal vesicle; Ut, uterus; Va, vagina; Vd, vas deferens. Bar = 1 mm; insert = 500 μm

**Fig. 4:**
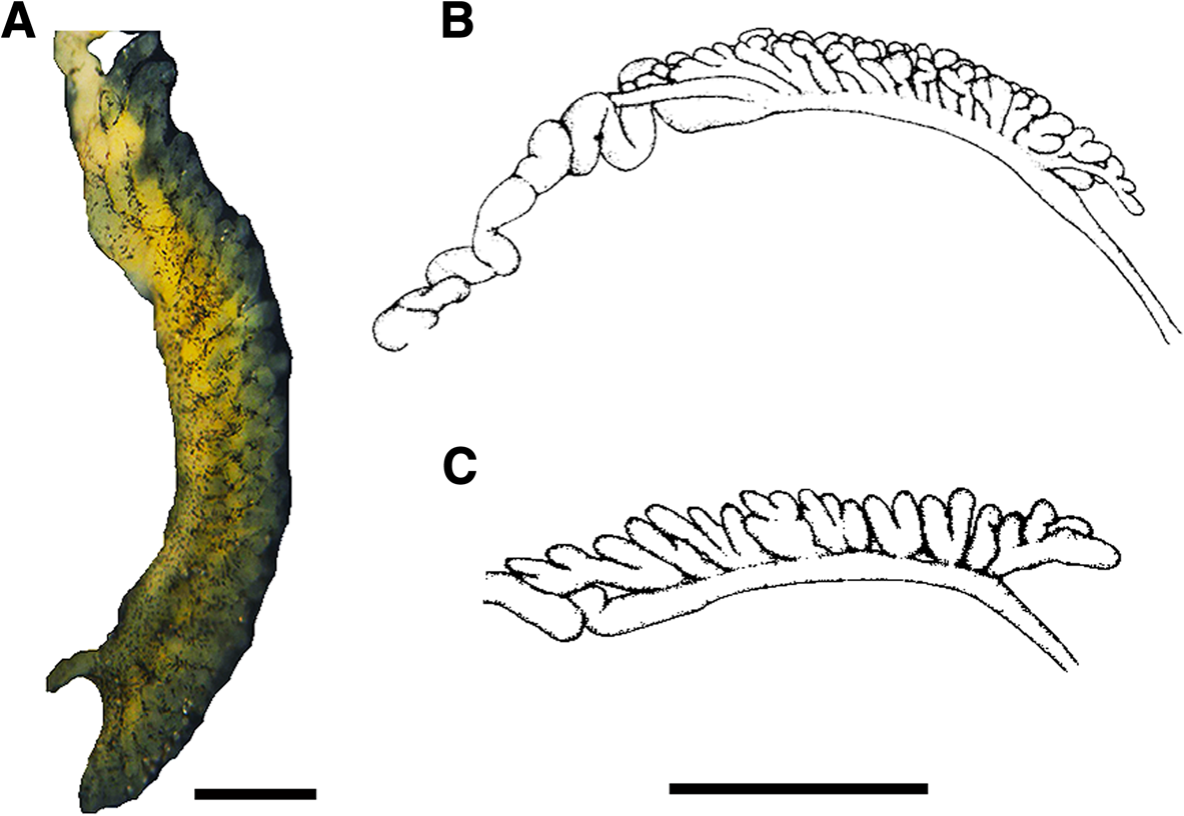
Prostate diverticula of *Biomphalaria straminea* from Tuyang, Shenzhen, Guangdong, China (**a**) *B. straminea* from Tangará da Serra, Mato Grosso, Brazil (**b**) and *B. kuhniana* from Tucurui, Tocantins river, Pará, Brazil (**c**). Both (**b**) and (**c**) are modified from Paraense [28]. Bar = 200 μm in (**a**) and 1 mm in (**b**) & (**c**)

Similar to the external morphology of the shell, the internal anatomical features of the collected snails, as inspected by careful and precise dissection, did not show significant variability among locations. The general feature of the reproductive system revealed many constant characteristics across the collected *Biomphalaria* populations. For example, the corrugation of vaginal wall is conspicuous; the ovotestis diverticula were short, unbranched; and the seminal vesicle had apparent diverticula. The spermatheca was found round or oval in shape. The prostate contained from 10 to 22 branched diverticula. The spermiduct wrinkle is very prominent. The penis sheath was longer than the preputium in all populations (Fig. 3). The ratio between penis sheath length to preputium length was the highest in Jinkui snail’s population with 1.41 ± 0.28 mm and lowest in Shenzhen reservoir snails which measured 1.24 ± 0.08 mm. The situation was reversed regarding the number of prostate diverticula where Shenzhen Reservoir populations significantly differed from Jinkui populations, with a mean number of 20.6 ± 2.3 and 10.8 ± 0.83 diverticula, respectively (Table 3). The prostate diverticula of Guangdong populations of *Biomphalaria* were very much similar to those recorded for *B. straminea* rather than *B. kuhniana* from Brazil (Fig. 4).

### Molecular data

Few variations were observed in the sequences of COI and 16S rRNA mitochondrial DNA regions from the 10 samples from each location site. Analysis of each mitochondrial gene alone revealed four (GenBank accession numbers: MF491792 − MF491795, 557 bp) and three (GenBank accession numbers: MF491796 − MF491798, 358 bp) haplotypes of Chinese *Biomphalaria*, respectively for COI and 16S rRNA without including gaps. There were only five variable sites for COI and the haplotype diversity was 0.8333, while for 16S rRNA there were three variable sites with a haplotype diversity of 1.00. Using the combined data set, six haplotypes were identified from 60 sampled sequences (distribution of all haplotypes is presented in Table 4). The number of variable sites was seven and haplotypes diversity was 0.9333. Both COI and 16S rRNA haplotypes showed low genetic divergence. COI haplotypes showed 0.002–0.007 pairwise distance and the number of base substitutions per site based on averaging over all haplotypes sequence pairs was 0.005 ± 0.002 (as obtained by a bootstrap procedure of 1000 replicates). The nucleotide diversity was 0.004488 among COI haplotypes. For 16S rRNA, the pairwise distance was 0.001–0.005 and the overall pairwise distance was 0.003 ± 0.003 while the nucleotide diversity was 0.005587. The divergence between COI + 16S rRNA haplotypes obtained from analyzing the combined data set was 0.001–0.005 pairwise distance with an average of 0.003 ± 0.001. The mean corrected distance across the combined haplotypes data set as a whole was 0.3% divergence and the nucleotide diversity was 0.002794.

**Table 4:** Guangdong haplotypes of *Biomphalaria straminea* and their distribution across the collection sites

Dataset	Haplotype	Distribution
COI	H11	Donghu Park (1), Shahexi Road (2), Guihua Road (3), Hubin Road (4), Jinkui (5), Tuyang (6)
	H39	Guihua Road (3)
	H57	Jinkui (5)
	H64	Tuyang (6)
16S rRNA	H11	Donghu Park (1)
	H31	Donghu Park (1), Shahexi Road (2), Guihua Road (3), Hubin Road (4), Jinkui (5), Tuyang (6)
	H62	Tuyang (6)
COI + 16S rRNA	H12	Donghu Park (1),
	H39	Guihua Road (3)
	H41	Donghu Park (1), Shahexi Road (2), Guihua Road (3), Hubin Road (4), Jinkui (5), Tuyang (6)
	H57	Jinkui (5)
	H62	Tuyang (6)
	H64	Tuyang (6)

All the populations conformed to the assumptions of the neutral hypothesis, as tested by Tajima’s D and significance of Tajima’s test for neutrality based on the total number of mutations [43]. No population was found to deviate from the assumptions of normality using the three data sets. The D values were − 0.796844, − 0.897067 and − 1.390308, respectively, for COI, 16S rRNA and COI + 16S rRNA data sets.

The samples of *B. straminea*, *B. kuhniana* and *B. intermedia* (*B. straminea* complex) that were obtained from Brazil and Colombia were also analyzed for unique 16S rRNA haplotypes in order to be used in the phylogenetic analysis. These sequence haplotypes of *B. straminea* complex were deposited in GenBank (Table 5).

**Table 5:** Haplotypes of South American *Biomphalaria straminea* complex, their location of collection and GenBank accession numbers

Haplotype	Location	Isolate	Accession numbers
H5522	Angioquia/Colombia	BK5522	MF491799
H2249	Pernambuco/Brazil	BS2249	MF491800
H2248	Pernambuco/Brazil	BS2248	MF491801
H231	Minas Gerais/Brazil	BS231	MF491802
H230	Minas Gerais/Brazil	BS230	MF491803
H397	Para/Brazil	BS397	MF491804
H3363	Distrito Federal/Brazil	BS3363	MF491805
H2250	Pernambuco/Brazil	BS2250	MF491806
H1859	Minas Gerais/Brazil	BS1859	MF491807
H2161	São Paulo/Brazil	BI2161	MF491808
H163	Minas Gerais/Brazil	BI163	MF491809
H597	Minas Gerais/Brazil	BI597	MF491810
H2160	São Paulo/Brazil	BI2160	MF491811
H598	Minas Gerais/Brazil	BI598	MF491812
H599	Minas Gerais/Brazil	BI599	MF491813

**Fig. 5:**
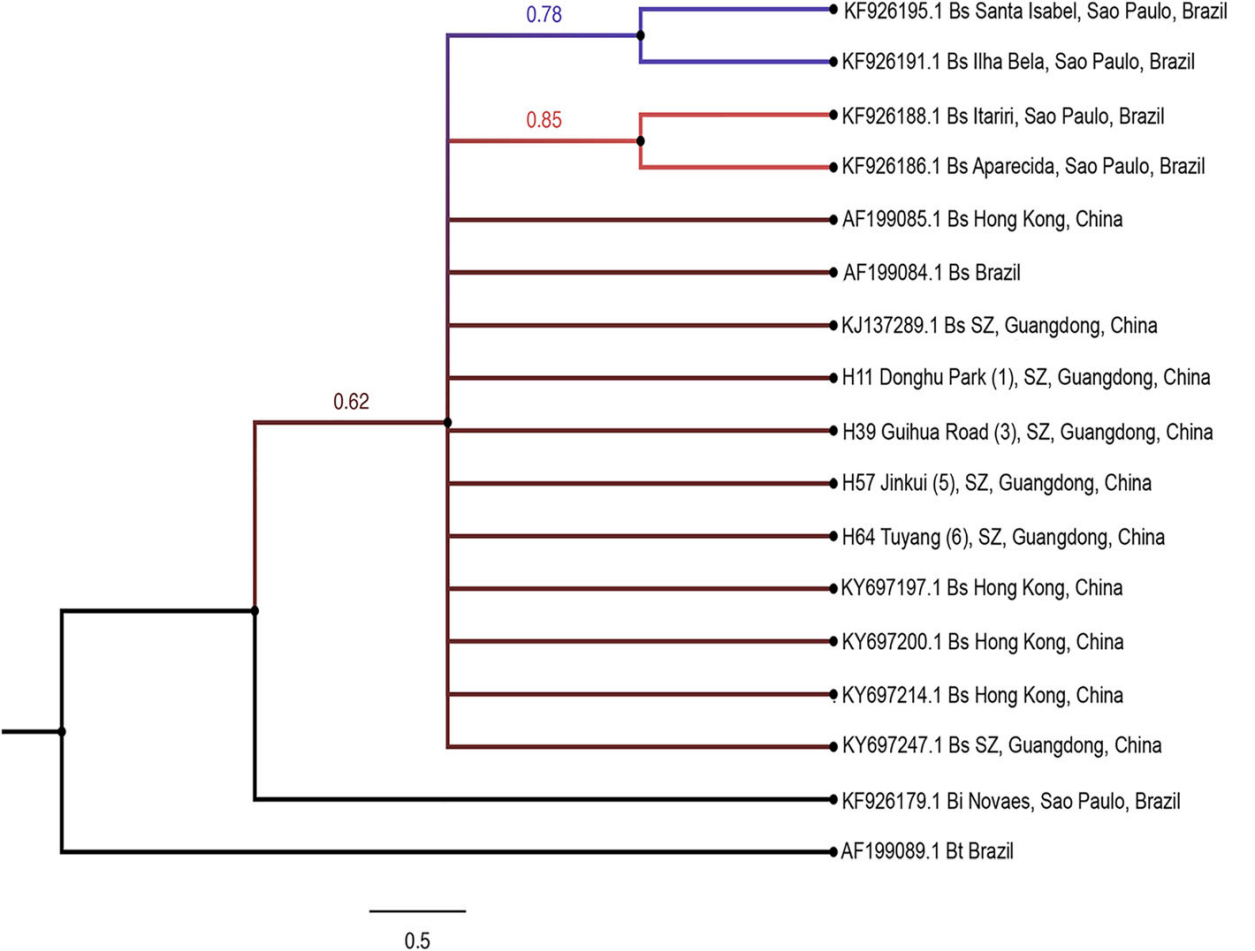
Bayesian phylogram based on analysis of COI sequences of Guangdong populations of *Biomphalaria straminea* (haplotypes) and Brazilian sequences retrieved from GenBank using *B. tenagophila* as an outgroup. Support values for individual branches are given as posterior probabilities based on 6 000 000 generations in a Bayesian analysis. Bs, *B. straminea*; Bt, *B. tenagophila*; SZ, Shenzhen

Phylogenetic reconstructions of COI haplotypes and GenBank sequences for *B. straminea* from Brazil and China using Bayesian inference with *B. tenagophila* (AF199089.1) as an outgroup resulted in a consensus tree with major nodal support values obtained based on the Bayesian posterior probabilities. The Guangdong populations and *B. straminea* from Hong Kong formed one monophyletic clade with a high nodal support indicating their close affinity to each other. In the same clade four sequences for *B. straminea* from São Paulo were also clustered with Chinese haplotypes. Basal to this clade situated *B. intermedia* from São Paulo with 0.62 nodal support value (Fig. 5).

**Fig. 6:**
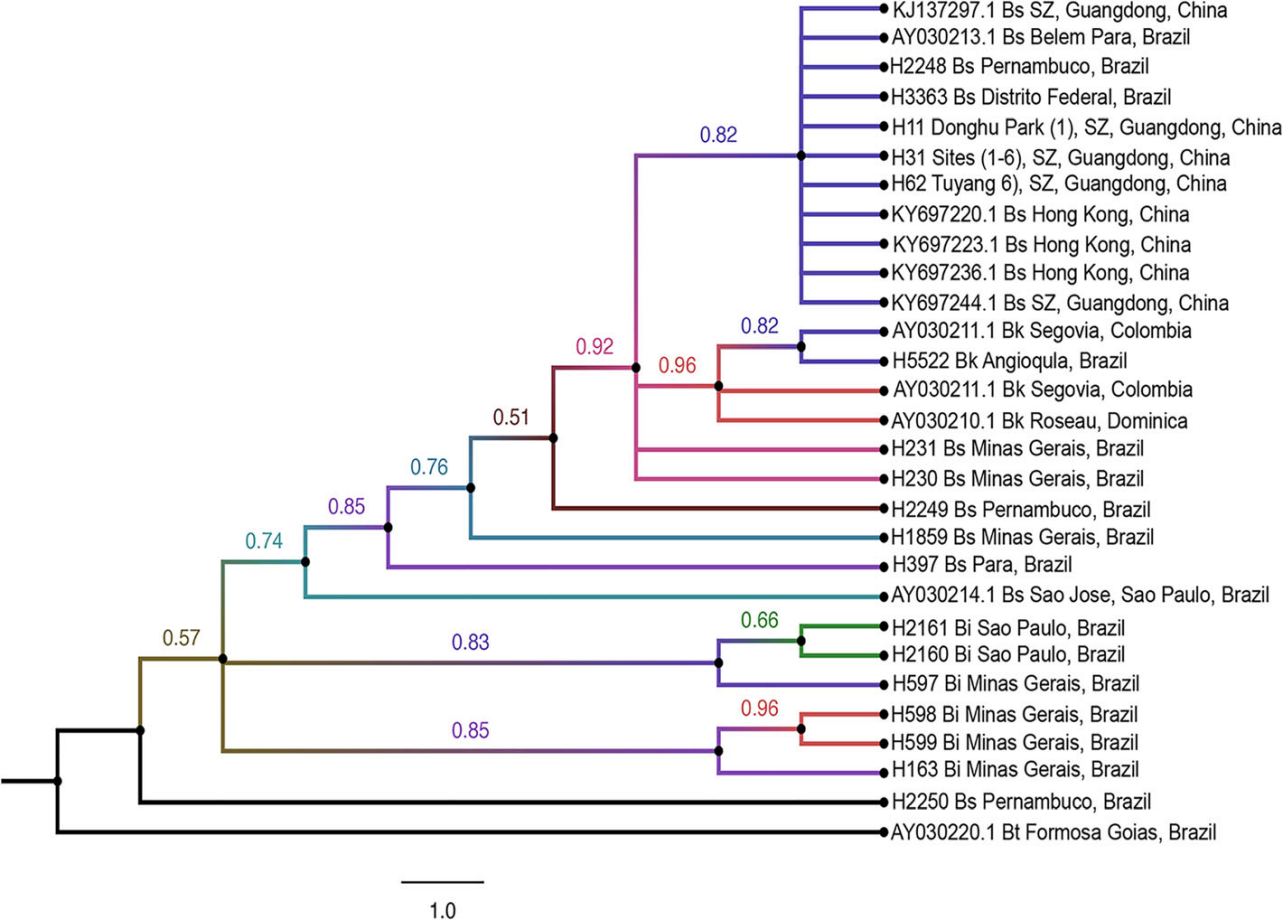
Bayesian phylogram based on analysis of 16 s rRNA data set for Guangdong populations of *Biomphalaria straminea* (haplotypes), South American samples from *B. straminea* complex (haplotypes) and reference sequences retrieved from GenBank using *B. tenagophila* as an outgroup. Support values for individual branches are given as posterior probabilities based on 6 000 000 generations in a Bayesian analysis. Bi, *B. intermedia*; Bk, *B. kuhniana*; Bs, *B. straminea*; Bt, B*. tenagophila*; SZ, Shenzhen

The analysis of 16S rRNA data set of Guangdong populations (haplotypes), South American samples of *B. straminea* complex (haplotypes) and reference sequences retrieved from GenBank using *B. tenagophila* as an outgroup showed the clustering of Guangdong populations in one clade with *B. straminea* sequences from Hong Kong of China and Brazil (Distrito Federal, Belem Para, and Pernambuco). Sequences of *B. straminea* from Minas Gerais were basal to this clade with a nodal support of 0.82 posterior probabilities. *Biomphalaria kuhniana* sequences formed one monophyletic group comprising samples from Brazil, Dominica and Colombia. Both of *B. straminea* and *B. kuhniana* groups described above shared a common ancestral sequence for *B. straminea* from Pernambuco, Brazil. *Biomphalaria intermedia* sequences clustered together in two monophyletic clades that were distal from Chinese *B. straminea* (Fig. 6).

**Fig. 7:**
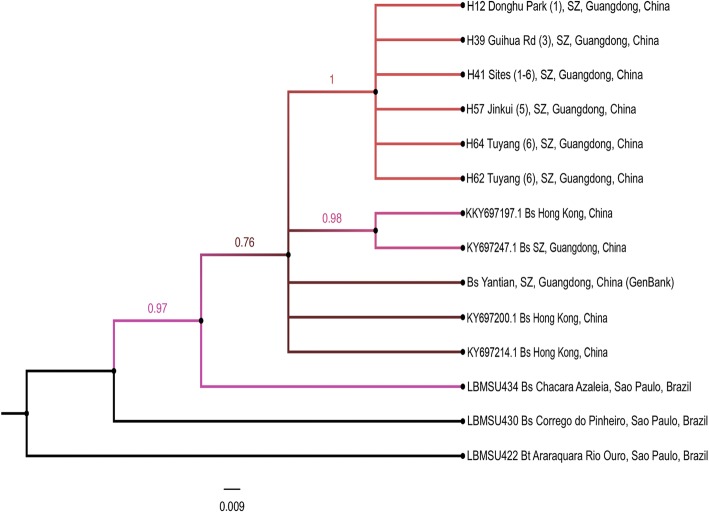
Bayesian phylogram based on analysis of combined COI and 16 s rRNA dataset for Guangdong populations of *Biomphalaria straminea* (haplotypes) and reference sequences retrieved from GenBank using *B. tenagophila* as an outgroup. Support values for individual branches are given as posterior probabilities based on 6 000 000 generations in a Bayesian analysis. Bs, *B. straminea*; Bt, *B. tenagophila*; SZ, Shenzhen

The analysis of the combined data sets (COI + 16S rRNA) showed a tree topology relatively similar to that obtained with COI data set. Guangdong populations were clustered in the same clade with *B. straminea* from Hong Kong and those previously identified from Yantian, Guangdong [22]. Basal to this clade was *B. straminea* from Chacara Azaleia, São Paulo, Brazil (LBMSU434) with strong nodal support > 0.70. Together the Guangdong populations from China and Brazilian isolates shares a common ancestor *B. straminea* from Corrego do Pinheiro, São Paulo, Brazil (LBMSU430). *B. tenagophila* (LBMSU422) from Araraquara, Rio Ouro, São Paulo was used as an outgroup (Fig. 7).

## Discussion

The present study attempted to identify the species of *Biomphalaria* snails introduced to southern China using morphological and molecular identification methods. The early reports on *Biomphalaria* invasion to Hong Kong identified the snails as *B. straminea* [14, 32, 44]. Subsequent invasions and spread were reported from other water bodies in southern China such as Guanlan and Dasha rivers in Shenzhen City, Guangdong Province’s rivers [18]. This distribution scheme led to the hypothesis of multiple invasions and colonization including different roots of introduction. Moreover, recent molecular investigation of several *Biomphalaria* populations from Guangdong indicated that most of the species currently harboring water systems are *B. kuhniana* not *B. straminea* [26]. This conclusion merited further careful investigation because *B. kuhniana* has so far been shown to be refractory to *S. mansoni* infection [27, 45], whereas *B. straminea* is generally considered to be a susceptible species, albeit in some cases at a low level, that can nonetheless transmit schistosomiasis [46–50], and even sustain natural transmission in some settings [13]. It is worth noting, however, that the taxonomic confusion within *Biomphalaria* has led some to speculate that naturally-infected *B. straminea* found in Venezuela may have in fact been *B. kuhniana* [13]*.* The fact remains that based on current data, *B. straminea* is generally considered to be of higher biomedical concern.

The identification of *Biomphalaria* was performed through morphological study of five specimens from each location sites in Shenzhen, Guangdong Province. Shell diameters ranged from 7.59 ± 0.45 mm as in Shenzhen reservoir population to 9.70 ± 0.93 mm in Guanlan River population with significant differences among the populations. According to Paraense [28], the average shell of adult *B. kuhniana* is smaller than 7.5 mm while that of *B. straminea* is from 11 mm to 16.5 mm. Although almost all of the current measurements are higher than 7.5 mm (the average diameter of *B. kuhniana*), which may support the snails to be *B. straminea*, the shell diameter is of limited value in discrimination between *B. straminea* and *B. kuhniana* due to the difficulty in recognizing full-grown shells in these species of planorbids [28]. Also, studies by Dupouy et al. [51] on the African pulmonate snail *Biomphalaria pfeifferi* (Krauss, 1848) concluded that environmental components may influence shell morphology through phenotypic versatility and/or through impacts of local natural selection. The differences observed in the shell size of *Biomphalaria* collected in this study could be attributed to environmental factors as well as the snails’ ages. The data from internal anatomy are considered more informative than external features. For example, the number of prostate diverticula varies significantly between *B. kuhniana* and *B. straminea*, with 4 to 9 observed in *B. kuhniana* versus 9 to 18 in *B. straminea* [28]*.* In the present study, in all the snails collected, the number of prostate diverticula ranged from 10.8 ± 0.83 to 20.6 ± 2.3 and the diverticula were long and branched. Similarly, the anatomy of reproductive organs of Hong Kong snails of *Biomphalaria* revealed an average number of prostate diverticula ranged from 11.67 to 17.67 [52] and both are close to the numbers recorded for South American *B. straminea* (9–18 prostate diverticula) [28]. The corrugations of vaginal wall and of the spermiduct are very prominent, similar to *B. straminea* from Brazil. The shape and number of diverticula in Guangdong populations supports its close affinity to *B. straminea* rather than *B. kuhniana* (Fig. 4).

Molecular based taxonomy of snail intermediate hosts has been developed to overcome erroneous species identification resulting from morphometric measurements. Mitochondrial data are reliable and informative and its consideration in snails taxonomy stems from the fact that the COI mitochondrial gene proved to be an ideal barcode locus and default marker adopted by the Consortium for the Barcode of Life for all groups of organisms [53, 54] including *Biomphalaria* spp. [55–58]. In the current study, molecular evidence, retrieved from sequencing two mitochondrial loci (COI and 16S rRNA), were in harmony with anatomical evidence and suggested a close affinity between Guangdong *Biomphalaria* and *B. straminea* from Hong Kong of China and Brazil as showed in the cladograms of the data sets analyzed. The absence of existing COI data for *B. kuhniana* limits its use as an independent identifier although the analysis of this locus in combination with 16S rRNA gave a quite similar tree topology as obtained with COI alone. Moreover, COI analysis excluded the possibility of Guangdong populations of *Biomphalaria* to be *B. intermedia*. The haplotypes from the present study were clustered in one clade with *B. straminea* sequences from Hong Kong, Guangdong of China and Brazil. *B. intermedia* originated from Brazil (KF926179.1 [59]) was situated in a different clade basal to the previous one with a nodal support of 0.62 posterior probabilities (Fig. 5). The phylogenetic tree in Fig. 5 showed that the COI sequences of the samples collected were identical to those of *B. straminea* from Brazil and those previously deposited in GenBank for Chinese *B. straminea* (Fig. 5). It is worthy to mention that looking for similar sequences for COI sequences deposited by Atwood et al. [27] for *B. kuhniana* (KJ137284 − KJ137287) using the BLAST tool showed that those sequences are 98–100% identical to *B. straminea* isolates of China and Brazil indicating that the authors may misidentified the samples collected. For 16S rRNA, enough data were available in GenBank records in addition to the sequences obtained from samples of *B. straminea* complex obtained from Brazil and Colombia. Guangdong haplotypes were clustered in the same clade with Hong Kong isolates of *B. straminea* (KY697220.1, KY697223.1 and KY697236.1 [52])*.* Both Guangdong and Hong Kong isolates from China were clustered with Brazilian samples of *B. straminea* from Distrito Federal and Pernambuco and one GenBank isolate from Belem Para, Brazil (AY030213.1) with a major nodal support values. *B. kuhniana* sequences represented an in-group clade with a nodal support of 0.96 posterior probabilities. This tree construction indicates that the Guangdong populations are in close affinity with *B. straminea* present in Hong Kong and both share South American ancestors of *B. straminea*. This finding is in accordance with Zeng et al. [52] who identified two phenotypes of *Biomphalaria* snails (black- and red-colored shell *Biomphalaria*) in sites located at the border between Hong Kong and Shenzhen (few miles away from the samples collected in our study) and in the New Territories in Hong Kong, including places adjacent to the mainland China (30–40 miles away from our samples). The two phenotypes were genetically indistinguishable and were similar to *B. straminea* abundant in mainland China and South America and suggested that the *B. straminea* snails in Hong Kong are South American in origin.

In the present study, both Guangdong populations from China and Brazilian *B. straminea* formed a clade of closely related species with a minimal genetic divergence between them (Figs. 5, 6 and 7). Moreover, the sequence divergence estimates (0.3%) and strong support for the relationship between these populations (as demonstrated by Bayesian posterior probability values) suggest that these are the same species, and closely related populations, rather than a separate species. Independent analyses of COI and 16S rRNA sequences showed low genetic variability with four and three distinct haplotypes, respectively for both genes, out of 60 sequences representing six different populations of *Biomphalaria* snails collected from Shenzhen. The low nucleotide diversity observed within the Guangdong populations (COI, 0.004488; 16S, 0.005587; COI + 16S rRNA, 0.002794) is consistent with the literature; in a recent study by Attwood et al. [26], the authors found three distinct COI sequences, with a nucleotide diversity of 0.00279, and only one unique 16S rRNA sequence.

The observed levels of genetic diversity across the Guangdong populations of *Biomphalaria*, suggests a more likely recent connection between these populations in view of the fast divergence of mitochondrial genes in pulmonates [60–62] which is expected in this recently introduced snail species. According to Attwood et al. [26], the observed divergence is relatively high given the small area of sampling, and they suggest that the diversity is not due to mutations occurring in Guangdong populations from China but rather was exhibited in ancestor Brazilian populations, indicating that the current Guangdong populations could represent multiple, independent, introductions followed by subsequent dispersal and colonization. This multiple colonization hypothesis is also supported by a previous study of 19 electrophoretlcally detected enzyme loci in four Hong Kong populations of *B. straminea* that showed high levels of variability among the populations. This variability resulted from multiple introduction events between 1973 and 1981 [44]. Although the relatively high genetic diversity at the COI (0.004488) locus, in comparison with other pulmonates, assumes divergence and mutations in ancestors’ snails at the introduction source, it can also be attributed to the reproductive traits of *B. straminea* and their high selfing rates. The presence of the populations in the same cladogram with *B. straminea* from Hong Kong (Fig. 5) may support the hypothesis that part of the snails currently distributed in Guangdong could be present as a result of peripheral dispersal of descendant snails from Hong Kong populations via passive transport or container ship traffic, or naturally due to connections between the water systems of the two adjacent localities. Finally, the genetic similarities among the snail populations strongly support the idea that these are derived from the original colonist populations via dispersal [63].

Preliminary experimental infection of filed captured-*B. straminea* from Shenzhen and their first generation with a Puerto Rican strain of *S. mansoni* showed, at different ratios of miracidial exposure, no infection in the exposed snails [64]. Although this finding suggests that the invasive *B. straminea* snails are incompatible with *S. mansoni* and thus there is a low risk of *S. mansoni* transmission in mainland China, it may not be appropriate to consider this as a final conclusion because the compatibility was tested with only one strain of *S. mansoni* from South America and the experiments were undertaken with the first generation of laboratory bred *B. straminea*. Further studies are needed to test the compatibility of different isolates of both Chinese *Biomphalaria* and *S. mansoni* from a variety of locations especially African strains because most of the imported cases of human infection are from Africa and the number is anticipated to increase due to the expansion in Chinese projects in Africa [23].

## Conclusions

The reassessment of the identity of *Biomphalaria* species present in southern China using morphological and molecular delimitation procedures suggests that *B. straminea* is the only *Biomphalaria* species abundant in southern China. However, to validate the current conclusion and in order to obtain a more reliable barcoding database for future studies, COI sequences from original reference snails (for *B. straminea* and *B. kuhniana*) from South America are required, and should be deposited in GenBank or other repositories. The current finding is important from a public health perspective due to the biomedical importance of *B. straminea* as an intermediate host for *Schistosoma mansoni,* and the potential risk of *Schistosoma mansoni* transmission to mainland China in view of the increase in labor services exchange with African countries where the parasite is endemic. More studies should be conducted to understand the biology of this snail and its competitive capacity with other local snail species and to describe its range of expansion. Moreover, the susceptibility of these local populations to *S. mansoni* should be tested. Better understanding of the population structure of this intermediate host snail is important to elucidate its potential role in schistosomiasis transmission, considering factors that may affect population structure such as environmental and demographical stability. Finally, it is widely encouraged to increase surveillance for all entry ports and checking for any exotic species, in order to reduce the likelihood of future introductions of potentially invasive species [64, 65].

## Additional file

Additional file 1: Multilingual abstracts in the five official working languages of the United Nations. (PDF 775 kb)

## References

[1] WHO (2011). Schistosomiasis. Fact Sheet number 115.

[2] Colley DG, Andros TS, Campbell CH (2017). Schistosomiasis is more prevalent than previously thought: what does it mean for public health goals, policies, strategies, guidelines and intervention programs?. Infect Dis Poverty.

[3] Brooker S (2002). Schistosomes, snails and satellites. Acta Trop.

[4] Diakite NR, Winkler MS, Coulibaly JT, Guindo-Coulibaly N, Utzinger J, N'Goran EK (2017). Dynamics of freshwater snails and *Schistosoma* infection prevalence in schoolchildren during the construction and operation of a multipurpose dam in central cote d'Ivoire. Infect Dis Poverty.

[5] Brown D (1994). Freshwater snails of Africa and their medical importance.

[6] Chitsulo L, Engels D, Montresor A, Savioli L (2000). The global status of schistosomiasis and its control. Acta Trop.

[7] Crompton D. How Much Helminthiasis Is There in the World? J Parasitol. 1999;85:397–403. 10386428

[8] Malek EA (1985). Snail hosts of schistosomiasis continued.

[9] Rollinson David (2010). Biomphalaria: Natural History, Ecology and Schistosome Transmission. Biomphalaria Snails and Larval Trematodes.

[10] Collins C, Xu J, Tang S (2012). Schistosomiasis control and the health system in P. R. China. Infect Dis Poverty.

[11] Teles HMS (2005). Distribuição geográfica das espécies dos caramujos transmissores de *Schistosoma mansoni* no Estado de São Paulo. Rev Soc Bras Med Trop.

[12] Woodruff DS, Mulvey M (1997). Neotropical schistosomiasis: African affinities of the host snail *Biomphalaria glabrata* (Gastropoda: Planorbidae). Biol J Linn Soc.

[13] DeJong RJ, Morgan JA, Paraense WL, Pointier JP, Amarista M, Ayeh-Kumi PF (2001). Evolutionary relationships and biogeography of *Biomphalaria* (Gastropoda: Planorbidae) with implications regarding its role as host of the human bloodfluke, *Schistosoma mansoni*. Mol Biol Evol.

[14] Meier-Brook C (1974). A snail intermediate host of *Schistosoma mansoni* introduced into Hong Kong. Bull World Health Organ.

[15] Pointier J, David P, Jarne P (2005). Biological invasions: the case of planorbid snails. J Helminthol.

[16] Zheng Q, Vanderslott S, Jiang B, Xu LL, Liu CS, Huo LL (2013). Research gaps for three main tropical diseases in the People’s Republic of China. Infect Dis Poverty.

[17] Liu Y, Huang Y, Zhuang W (1982). The discovery of *Biomphalaria straminea* (dunker), an intermediate host of *Schistosoma mansoni,* from China. Acta Zootaxon Sin.

[18] Pan S, Chen P, Rong S, Liu J, Wang J, Chen Z, Zhong J (1993). Investigation on *Biomphalaria straminea,* an intermediate host of *Schistosoma mansoni* in Shenzhen City. South Chin J Prev Med.

[19] Gao S, Li X, Huang S, Xie X, Mei S, Ruan C, Huang D (2013). Primary investigation of distribution and ecological environment of *Biomphalaria straminea* in Dasha and Guanlan rivers in Shenzhen areas. Chin Trop Med.

[20] Houghton JT, Ding Y, Griggs DJ, Noguer M, van der Linden PJ, Dai X (2001). Climate change 2001: the scientific basis.

[21] Zhou XN, Yang GJ, Yang K, Wang XH, Hong QB, Sun LP (2008). Potential impact of climate change on schistosomiasis transmission in China. Amer J Trop Med Hyg.

[22] Paraense W, Cunha A (1970). Planorbídeos hospedeiros intermediários do *Schistosoma mansoni*. Esquistossomose mansoni.

[23] Wang W, Liang YS, Hong QB, Dai JR (2013). African schistosomiasis in mainland China: risk of transmission and countermeasures to tackle the risk. Parasit Vectors.

[24] Lu XT, Gu QY, Limpanont Y, Song LG, Wu ZD, Okanurak K (2018). Snail-borne parasitic diseases: an update on global epidemiological distribution, transmission interruption and control methods. Infect Dis Poverty.

[25] Teodoro TM, Janotti-Passos LK, dos Santos Carvalho O, Caldeira RL (2010). Occurrence of *Biomphalaria cousini* (Mollusca: Gastropoda) in Brazil and its susceptibility to *Schistosoma mansoni* (Platyhelminths: Trematoda). Mol Phylog Evol.

[26] Attwood SW, Huo GN, Qiu JW (2015). Update on the distribution and phylogenetics of *Biomphalaria* (Gastropoda: Planorbidae) populations in Guangdong Province. China Acta Trop.

[27] Paraense WL (2001). The schistosome vectors in the Americas. Mem Inst Oswald Cruz.

[28] Paraense WL (1988). *Biomphalaria kuhniana* (Clessin, 1883), planorbid mollusc from South America. Mem Inst Oswald Cruz.

[29] WHO (1965). Snail control in the prevention of Bilhariziasis. Ser No 50 (Geneva, 1965).

[30] Mandahl-Barth G (1957). Intermediate hosts of *Schistosoma*: African *Biomphalaria* and *Bulinus*: II. Bull World Health Organ.

[31] PAHO. A Guide for the Identification of the Snail Intermediate Hosts of Schistosomiasis in the Americas. Scientific. vol. Publication No. 168: Pan American Health Organization, Regional Office of the World Health Organization Washington, DC; 1968.

[32] Yipp MW (1990). Distribution of the schistosome vector snail, *Biomphalaria straminea* (Pulmonata: Planorbidae) in Hong Kong. J Molluscan Stud.

[33] Madsen H (1990). Biological methods for the control of freshwater snails. Parasitol Today.

[34] Pointier JP, Theron A, Borel G (1993). Ecology of the introduced snail *Melanoides tuberculata* (Gastropoda: Thiaridae) in relation to *Biomphalaria glabrata* in the marshy forest zone of Guadeloupe, French West Indies. J Moll Stud.

[35] Scheffé H (1959). The analysis of variance.

[36] Bonnaud L, Boucher-Rodoni R, Monnerot M (1994). Phylogeny of decapod cephalopods based on partial 16S rDNA nucleotide sequences. C R Acad Sci III.

[37] Vrijenhoek R (1994). DNA primers for amplification of mitochondrial cytochrome c oxidase subunit I from diverse metazoan invertebrates. Mol Marine Biol Biotechnol.

[38] Hall TA (1999). BioEdit: a user-friendly biological sequence alignment editor and analysis program for windows 95/98/NT. Nucleic acids symposium series.

[39] Thompson J, Higgins D, Gibson T (1997). CLUSTAL X multiple sequence alignment program.

[40] Ronquist F, Huelsenbeck JP (2003). MrBayes 3: Bayesian phylogenetic inference under mixed models. Bioinformatics.

[41] Posada D (2008). jModelTest: phylogenetic model averaging. Mol Biol Evol.

[42] Tamura K, Stecher G, Peterson D, Filipski A, Kumar S (2013). MEGA6: molecular evolutionary genetics analysis version 6.0. Mol Biol Evol.

[43] Tajima F (1989). Statistical method for testing the neutral mutation hypothesis by DNA polymorphism. Genetics.

[44] Woodruff DS, Mulvey M, Yipp MW (1985). Population genetics of *Biomphalaria straminea* in Hong Kong. A neotropical schistosome-transmitting snail recently introduced into China. J Hered.

[45] Teodoro TM, Jannotti-Passos LK, OdS C, Grijalva MJ, Baús EG, Caldeira RL (2011). Hybridism between *Biomphalaria cousini* and *Biomphalaria amazonica* and its susceptibility to *Schistosoma mansoni*. Mem Inst Oswaldo Cruz.

[46] Barboza DM, Zhang C, Santos NC, Silva MMBL, Rollemberg CVV, de Amorim FJR (2012). *Biomphalaria* species distribution and its effect on human *Schistosoma mansoni* infection in an irrigated area used for rice cultivation in Northeast Brazil. Geospat Health.

[47] Fernandez MA, Pieri OS (2001). Infection by *Schistosoma mansoni* Sambon 1907 in the first four months of life of *Biomphalaria straminea* (dunker, 1848) in Brazil. Mem Inst Oswaldo Cruz.

[48] Fernandez MA, Thiengo SC (2002). Susceptibility of *Biomphalaria straminea* (dunker, 1848) from Serra da Mesa dam, Goiás, Brazil to infection with three strains of *Schistosoma mansoni* Sambon, 1907. Mem Inst Oswaldo Cruz.

[49] Souza C, MdS R, Azevedo M, Araújo N (1981). Susceptibility of populations of biomphalaria straminea (dunker, 1848) from Minas Gerais, to *Schistosoma mansoni* infection. Rev Inst Med Trop Sao Paulo.

[50] Fernandez MA, Thiengo SC (2010). Susceptibility of *Biomphalaria straminea* from Peixe Angical dam, Tocantins, Brazil to infection with three strains of *Schistosoma mansoni*. Mem Inst Oswaldo Cruz.

[51] Dupouy J, Rousseau D, Dussart G, Liaud MV, Nassi H (1993). Correspondence analysis of shell morphology in the African freshwater snail *Biomphalaria pfeifferi* (Kraus 1848) (Pulmonata: Gastropoda). Biol J Linn Soc.

[52] Zeng X, Yiu WC, Cheung KH, Yip HY, Nong W, He P, Yuan D, Rollinson D, Qiu J-W, Fung MC (2017). Distribution and current infection status of *Biomphalaria straminea* in Hong Kong. Parasit Vectors.

[53] Hebert PD, Ratnasingham S, de Waard JR (2003). Barcoding animal life: cytochrome c oxidase subunit 1 divergences among closely related species. Proc Biol Sci.

[54] Schindel DE, Miller SE (2005). DNA barcoding a useful tool for taxonomists. Nature.

[55] Jørgensen A, Kristensen TK, Stothard JR (2007). Phylogeny and biogeography of African *Biomphalaria* (Gastropoda: Planorbidae), with emphasis on endemic species of the great east African lakes. Zool J Linnean Soc.

[56] Standley C, Pointier J, Issia L, Wisnivesky-Colli C, Stothard J (2011). Identification and characterization of *Biomphalaria peregrina* (Orbignyi, 1835) from Agua Escondida in northern Patagonia, Argentina. J Nat Hist.

[57] Collado GA, Vila I, Méndez MA (2011). Monophyly, candidate species and vicariance in *Biomphalaria* snails (Mollusca: Planorbidae) from the southern Andean Altiplano. Zool Scripta.

[58] Campbell G, Jones CS, Lockyer AE, Hughes S, Brown D, Noble LR, Rollinson D (2000). Molecular evidence supports an African affinity of the Neotropical freshwater gastropod, *Biomphalaria glabrata*, say 1818, an intermediate host for *Schistosoma mansoni*. Proc Biol Sci.

[59] Palasio RGS, de Almeida Guimarães MC, Ohlweiler FP, Tuan R (2017). Molecular and morphological identification of *Biomphalaria* species from the state of São Paulo. Brazil ZooKeys.

[60] Yamazaki N, Ueshima R, Terrett JA, Yokobori S-i, Kaifu M, Segawa R (1997). Evolution of pulmonate gastropod mitochondrial genomes: comparisons of gene organizations of Euhadra, Cepaea and Albinaria and implications of unusual tRNA secondary structures. Genetics.

[61] Weaver KF, Anderson T, Guralnick R (2006). Combining phylogenetic and ecological niche modeling approaches to determine distribution and historical biogeography of Black Hills mountain snails (Oreohelicidae). Diversit Distrib.

[62] Wethington AR, Lydeard C (2007). A molecular phylogeny of Physidae (Gastropoda: Basommatophora) based on mitochondrial DNA sequences. J Molluscan Stud.

[63] Mohammed NA, Madsen H, Ahmed AA (2016). Types of trematodes infecting freshwater snails found in irrigation canals in the East Nile locality, Khartoum, Sudan. Infect Dis Poverty.

[64] Qu G, Wang W, Lu X, Dai J, Li X, Liang Y (2016). Evaluating the risk of *Schistosoma mansoni* transmission in mainland China. Parasitol Res.

[65] Stanton MC, Adriko M, Arinaitwe M, Howell A, Davies J, Allison G (2017). Intestinal schistosomiasis in Uganda at high altitude (>1400 m): malacological and epidemiological surveys on mount Elgon and in Fort Portal crater lakes reveal extra preventive chemotherapy needs. Infect Dis Poverty.

