# Supplementary material for: Morphological and molecular characterization of invasive Biomphalaria straminea in southern China
Source: Infect Dis Poverty. 2018 Dec 8;7:120. doi: 10.1186/s40249-018-0505-5 (PMC6286595; doi:10.1186/s40249-018-0505-5)
Supplement: Supplementary file 1 — Multilingual abstracts in the five official working languages of the United Nations. (PDF 775 kb) [file 40249_2018_505_MOESM1_ESM.pdf]

Translation of the abstract into the six official working languages of the United Nations

الخواص المورفولوجية والجزيئية لقواقع بيومفلاريا سترامينيا الدخيلة في جنوب الصين

محمد ر. حبيب, شان لوفي, يون-هاي جو, ون-بياو جو, كلير ج. ستانلي, روبيرتا ل. كالديرا, شياو-نونغ تشو

الملخص
الخلفية: مرض البلهارسيا هو مرض طفيلي شائع مصنف من قبل منظمه الصحة العالمية كواحد من الآمراض الإستوائية المهملة وتعتبر البهارسيا المعوية واحدة من اشكال المرض التي تسببها ديدان البلهارسية المانسونية التي تستخدم قوقع البيومفلاريا كعائل وسيط وقد قدمت البيومفلاريا إلى هونج كونج بالصين مع شحنات النباتات المائية القادمة من البرازيل وسرعان ما أسست بيئاتها في جنوب الصين وأكدت الدراسات السابقة أن هذه القواقع الدخيلة علي الصين هي من النوع بيومفلاريا سترامينيا التي تعد أحد الأنواع الناقلة للبلهارسيا المعوية في أمريكا الجنوبية إلا أن الدراسات الأكثر حداثة قد أشارت الي وجود نوع أخر من القواقع وهو البيومفلاريا كونيانا التي تنتشر أيضا في أمريكا الجنوبية ولكنها ليست قابلة للعدوي بطفيل البلهارسا وبالتالي ليس لها دور في إنتقال المرض وعلي هذا النحو فإنه من المهم التعرف بدقه علي أنواع القواقع المنتشرة حاليا في جنوب الصين وخاصه في ظل التقارير المستجدة عن إصابات البلهارسيا التي تصيب العمال الصينيين العائدين من افريقيا.
الأساليب: لقد إستخدمنا أساليب التعرف المورفولوجية والجزيئية وذلك لكي نحد بدقة نوع قواقع البيومفلاريا المنتشرة في مقاطعه قوانغدونغ في جنوب الصين ولكي نفهم بوضوح الصورة الجزيئية لأنواع القواقع قمنا بتحليل البيانات الجزيئية التي تم الحصول عليها من الميتوكندريا (جين السيتوكروم أوكسيداز ١ وجين ال ١٦ اس الريبوسومي) لستة مجموعات من قواقع البيومفلاريا من مدينة شنتشن في مقاطعه قوانغدونغ وبالإضافة إلى ذلك درسنا الشكل الخارجي للصدفة والتشريح الداخلي للأعضاء التناسلية للقواقع.
النتائج: أشارت كلا من الأدلة المورفولوجية والجزيئية إلى تقارب وثيق بين قواقع البيومفلاريا من قوانغدونغ وبين قواقع البيومفلاريا سترامينيا من البرازيل وكانت صدفة القواقع متطابقة تقريبا في جميع مجموعات البيومفلاريا التي جمعت حيث كانت لفات الصدفة مدوره علي جانب واحد وللأسفل من ناحية أخرى وذات سطح املس و فتحة صدفة علي شكل بيضاوي وذات سرة عميقة وفيما يتعلق بالتشريح الداخلي فقد رجح شكل وعدد رتوج البروستاتا (تراوحت من ١١,٦٧ إلي ١٧,٦٧) تقارب قواقع مقاطعه قوانغدونغ للبيومفلاريا سترامينيا عن بيومفلاريا كونيانا ولم يتعارض التحليل الجزيئي مع التحليل المورفولوجي حيث لوحظ تمايز وراثي صغير بين مجموعات القواقع التي تم تجميعها وبإستخدام تحليل التقارب الجيني للتتابعات الجينية للأنماط الفردانية لجين السيتوكروم أوكسيداز ١ وجين ال ١٦ اس الريبوسومي من القواقع التي تم جمعها ولقواقع بيومفلاريا سترامينيا من الصين والبرازيل أن مجموعات قواقع البيومفلاريا من قوانغدونغ تواجدت في فرع حيوي واحد مع بيومفلاريا سترامينيا من هونج كونج والبرازيل في إشارة إلى تقاربها الوثيق مع بعضها البعض.
الاستنتاجات: بينت البيانات التي تم الحصول عليها في الدراسة الحالية ان مجموعات قواقع البيومفلاريا التي تم دراستها من قوانغدونغ هي من النوع بيومفلاريا سترامينيا ونحن نفترض ان تلك القواقع اما قدمت عن طريق الإنتشار الغير مباشر من هونج كونج أو نتيجة طرق دخول متعددة من البرازيل.

Translated from English version into Arabic by Mohamed R. Habib

中国南部入侵蒿杆双脐螺的形态学与分子特征研究

Mohamed R. Habib, 吕山, 郭云海, 顾文彪, Claire J. Standley, Roberta L. Caldeira, 周晓农

摘要
引言：血吸虫病是一种被世界卫生组织定义为被忽视热带病的常见寄生虫病。曼氏血吸虫病是由曼氏血吸虫寄生于人体而引起的疾病，通过中间宿主双脐螺传播。双脐螺随着船运的水生植物从巴西侵入香港，并很快在中国南部建立了孳生地。早期研究显示，入侵的双脐螺为蒿杆双脐螺，是南美地区传播曼氏血吸虫的适宜宿主。然而，近期分子研究表明还有另一种南美地区的螺种存在，即库恩双脐螺，该螺对血吸虫不易感。因此，准确鉴别中国南方分布的双脐螺螺种非常重要，特别是中国赴非洲务工人员罹患曼氏血吸虫病屡见报道。
方法：本研究同时采用形态学和分子分类学工具来鉴定中国广东省双脐螺螺种。为细致了解该螺种的分子背景，使用来自广东省深圳市 6 个双脐螺种群的线粒体 DNA 数据（CO I 和 16S rRNA 序列）建立了系统发育树。同时，比较研究了这些螺的螺壳外部形态和生殖系统的内部解剖结构。
结果：形态学和分子学证据均表明广东双脐螺种群与巴西的蒿杆双脐螺相似性很高。所有种群的螺壳形态指标基本一致，其螺旋在一侧为圆形，另一侧呈亚梭角，边缘光滑，卵圆形的壳口弯向一侧，有一个较深的脐。广东种群的前裂腺囊形态和数量（11.67−17.67），支持其为蒿杆双脐螺，而非库恩双脐螺。分子特征与形态学分析结果一致。各种群间的遗传差异较小。基于本研究所得 CO I 和 16S rRNA 单倍型以及来自巴西和中国蒿杆双脐螺相关序列建立贝叶斯模型进行系统发育分析，结果显示，中国广东、香港种群与巴西蒿杆双脐螺聚在一个分支中，提示它们的相似性极高。
结论：本研究采集的双脐螺为蒿杆双脐螺，推测这些种群或来自香港的近距离被动扩散，或来自巴西远距离的多次迁入。

Translated from English version into Chinese by Xiao-Nong Zhou

Caractérisation morphologique et moléculaire du gastéropode invasif *Biomphalaria straminea* en Chine du Sud

Mohamed R. Habib, Shan Lv, Yun-Hai Guo, Wen-Biao Gu, Claire J. Standley, Roberta L. Caldeira, Xiao-Nong Zhou

Résumé

Contexte: La schistosomiase est une maladie parasitaire courante, désignée comme maladie tropicale négligée par l'Organisation mondiale de la santé. La schistosomiase à S. mansoni est une forme de la maladie causée par le trématode digène *Schistosoma mansoni*, transmise par un hôte intermédiaire du genre *Biomphalaria* spp. *Biomphalaria* a été introduit à Hong Kong, en Chine, dans des envois de plantes aquatiques en provenance du Brésil. Il s'est installé dans le sud du pays. Des études antérieures de *Biomphalaria* spp. introduits dans le sud de la Chine ont identifié les escargots comme *Biomphalaria straminea*, une espèce sensible impliquée dans la transmission de *S. mansoni* en Amérique du Sud. Cependant, des études moléculaires récentes ont également indiqué la présence d'une autre espèce sud-américaine, *B. kuhniana*, qui est réfractaire à l'infestation. Il est donc important d'identifier avec précision les espèces actuellement présentes dans le sud de la Chine, en particulier avec les nouveaux signalements d'infestation par *S. mansoni* de travailleurs chinois revenant d'Afrique.

Méthodes: Nous avons combiné des outils morphologiques et de taxonomie moléculaire pour identifier précisément la distribution de *Biomphalaria* spp. dans la province de Guangdong, en Chine du Sud. Afin de déterminer clairement le profil moléculaire de l'espèce, nous avons construit une phylogénie en utilisant des données d'ADNmt (séquences d'ADNmt et d'ARNr 16S) de six populations de *Biomphalaria* spp. provenant de la ville de Shenzhen, dans la province de Guangdong. De plus, nous avons examiné la morphologie externe de la coquille et l'anatomie interne des organes reproducteurs.

Résultats: Les données morphologiques et moléculaires indiquent une affinité proche entre les populations de *Biomphalaria* spp. du Guangdong et *B. straminea* du Brésil. La morphologie de la coquille était approximativement identique dans toutes les populations prélevées, avec des verticilles arrondis d'un côté et sous-angulés de l'autre, une périphérie lisse, une ouverture ovoïde incurvée d'un côté et un ombilic profond. La forme et le nombre de diverticules prostatiques (variant de 11,67 à 17,67) dans les populations du Guangdong confirment l'affinité étroite avec *B. straminea* plutôt que *B. kuhniana*.L'analyse moléculaire ne contredit pas l'analyse morphologique. Une faible différenciation génétique a été observée dans les populations prélevées de *Biomphalaria*. L'analyse phylogénétique des haplotypes d'ACO et d'ARNr 16S d'escargots prélevés et des séquences de *B. straminea* du Brésil et de Chine, sur la base des inférences bayésiennes, révèle que les populations du Guangdong se retrouvent dans le même clade que *B. straminea* de Hong Kong en Chine et *B. straminea* du Brésil, ce qui révèle leur étroite affinité.

Conclusions: Les données obtenues dans la présente étude montrent clairement que les populations de *Biomphalaria* spp. examinées appartiennent à l'espèce *B. straminea*, et nous supposons que ces escargots ont été introduits par dispersion passive depuis Hong Kong ou à la suite de multiples introductions en provenance du Brésil.

Translated from English version into French by Gabriela Kouahla, proofread by Suzanne Assenat, through

TRANSLATORS
WITHOUT BORDERS

Морфологическая и молекулярная характеризация инвазивного вида *biomphalaria straminea* в южном Китае

Мохамед Р. Хабиб, Шань Лв, Юнь-Хай Го, Вэнь-Бяо Гу, Клэр Дж. Стендли, Роберта Л. Калдейра, Сяо-Нун Чжоу

Аннотация

Справочная информация: Шистосомоз является распространенным паразитарным заболеванием, которое Всемирная организация здравоохранения относит к забытым тропическим болезням. Шистосомоз Мансони — это разновидность болезни, вызываемая трематодой *Schistosoma mansoni*, которая передается через промежуточного хозяина видов *Biomphalaria* . Улитки *Biomphalaria*были завезены в Гонконг, Китай при поставке водных растений из Бразилии, и они быстро прижились в южном Китае. В ранних исследованиях улитки видов *Biomphalaria*, завезённых в южный Китай, были идентифицировали как *Biomphalaria straminea* – один из видов, который считается восприимчивыми к

передаче *S. mansoni* в Южной Америке. Однако недавние молекулярные изучения также показали присутствие другого южноамериканского вида *B. kuhniana,* который является устойчивым к инфицированию. В этой связи важно правильно идентифицировать виды, в настоящее время распространенные в южном Китае, особенно в связи с появляющимися сообщениями об инфекции *S. mansoni* у китайских рабочих, возвращающихся из Африки.
Методы: Для точного определения улиток видов *Biomphalaria,* распространенного в провинции Гуандун в южном Китае, мы применили сочетание морфологических и молекулярных инструментов таксономии. Чтобы достичь ясности в понимании молекулярного профиля данных видов, мы построили филогению при помощи данных mtDNA (последовательностей COI и 16S rRNA) из шести популяций видов *Biomphalaria* по городу Шэньчжэнь провинции Гуандун. Кроме того, мы обследовали внешнюю морфологию раковины и внутреннюю анатомию репродуктивных органов.
Результаты: Как морфологические, так и молекулярные данные указывают на близкое сходство между популяциями видов *Biomphalaria* из Гуандуна и *B. straminea* из Бразилии. У всех собранных популяций морфология раковин была примерно одинаковой с закругленными завитками с одной стороны и угловатыми с другой стороны; с гладкой периферией; яйцеобразным открытием, наклоненным в одну сторону и глубоким центральным выступом. Форма и величина выпячивания предстательной железы (в диапазоне от 11,67 до 17,67) у популяций в Гуандуне поддерживает предположение об их близком сходстве скорее с *B. straminea,* чем с *B. kuhniana.* Молекулярный анализ не противоречил морфологическому анализу. По собранным популяциям *Biomphalaria* было замечено незначительное генетическое различие. Филогенетический анализ гаплотипов COI и 16S rRNA у собранных улиток и последовательности *B. straminea* из Бразилии и Китая, выполненные с использованием Байесовского вывода, показал, что популяции Гуандуна были сгруппированы в одной филогенетической ветви с *B. straminea* из Гонконга в Китае и с *B. straminea* из Бразилии, что указывает на их родство друг с другом.
Выводы: Данные, полученные в настоящем исследовании, со всей очевидностью показывают, что популяции изученных видов *Biomphalaria* принадлежат к виду *B. straminea,* и мы полагаем, что эти улитки появились в результате пассивного распространения из Гонконга в Китае или были многократно завезены из Бразилии.

Translated from English version into Russian by Natalia Potashnik, proofread by Liudmila Tomanek, through

Caracterización morfológica y molecular de la *Biomphalaria straminea* invasiva en el sur de China

Mohamed R. Habib, Shan Lv, Yun-Hai Guo, Wen-Biao Gu, Claire J. Standley, Roberta L. Caldeira, Xiao-Nong Zhou

Resumen
Antecedentes: La esquistosomiasis es una enfermedad común parasitaria considerada como una enfermedad tropical desatendida según la Organización Mundial de la Salud. La esquistosomiasis mansoni es una variante de la enfermedad causada por la trematoda digenea *Schistosoma mansoni*, que se transmite por la *Biomphalaria* spp. como huésped intermediario. La*Biomphalaria* se introdujo en Hong Kong, China, mediante envíos de plantas acuáticas desde Brasil, y los caracoles se asentaron rápidamente en el sur de China. Anteriores estudios sobre la *Biomphalaria* spp. introducida en el sur de China identificaron a los caracoles como *Biomphalaria straminea*, una de las especies posiblemente implicadas en la transmisión del *S. mansoni* en América del Sur. No obstante, investigaciones moleculares recientes también señalaron la presencia de otra especie procedente de América del Sur, la *B. kuhniana,* refrectaria a la infección. Por todo ello, es importante identificar de manera adecuada las especies dispersas por el sur de China, especialmente las que cuentan con avisos recientes de*infecciones*de S. mansoniactivas en trabajadores chinos que regresan de África.
Metodología: Hemos combinado herramientas de taxonomía morfológica y molecular para identificar de manera precisa la *Biomphalaria* spp. dispersa en la provincia de Guandong, en el sur de China. Con el fin de entender correctamente el perfil molecular de las especies, hemos elaborado un árbol filogénico empleando ADNmt (COI y secuencias rRNA) de seis poblaciones de *Biomphalaria* spp. de la ciudad de Shenzhen, en la provincia de Guandong. Además, hemos examinado la morfología externa de la concha y la anatomía interna de los órganos reproductores.
Resultados: Las pruebas morfológicas y moleculares señalaron una afinidad cercana entre las poblaciones de *Biomphalaria* spp. de Guangdong y las de*B. straminea* de Brasil. La morfología de la concha es prácticamente idéntica en todas las poblaciones estudiadas, con espirales circulares en un lado y subanguladas en el otro, con

bordes delicados, una apertura con forma de huevo inclinada hacia un lado y un umbilico profundo. El número y la forma de los divertículos prostáticos (que oscila de 11,67 a 17,67) en las poblaciones de Guangdong avalan la afinidad con la *B. straminea* más que con la *B. kuhniana.* Los análisis moleculares no entraron en conflicto con los análisis morfológicos. Advertimos una pequeña diferenciación genética en las poblaciones de *Biomphalaria* recopiladas. Los análisis filogenéticos de haplotipos de COI y ARNr 16S extraídos de los caracoles recogidos y de secuencias de *B. straminea* de Brasil y China mediante inferencia bayesiana revelaron que las poblaciones de Guangdong fueron agrupadas en un clado con *B. straminea* de Hong Kong y *B. straminea* de Brasil, mostrando así la afinidad cercana entre ambas.
Conclusiones: Los datos obtenidos del presente estudio muestran claramente que las poblaciones de *Biomphalaria* spp. investigadas son *B. straminea* y asumimos por tanto que estos caracoles fueron introducidos o bien de manera pasiva y dispersa desde Hong Kong o como resultado de múltiples rutas de introducción desde Brasil.

Translated from English version into Spanish by David López Canelada, proofread by Estefanía Sosa Albert, through

TRANSLATORS
WITHOUT BORDERS
